# Unveiling the digital future: perspectives of Hungarian physicians under 35 years old on eHealth solutions

**DOI:** 10.3389/fdgth.2024.1464642

**Published:** 2025-01-27

**Authors:** Zsuzsa Győrffy, Bence Döbrössy, Julianna Boros, Edmond Girasek

**Affiliations:** ^1^ Faculty of Medicine, Institute of Behavioral Sciences, Semmelweis University, Budapest, Hungary; ^2^ Department of Family Medicine, Faculty of Medicine, Semmelweis University, Budapest, Hungary; ^3^ Hungarian Demographic Research Institute, Budapest, Hungary

**Keywords:** digital health, young doctors, adherence, paradigm shift, participatory medicine, e-physcians, burnout

## Abstract

**Background:**

The COVID-19 pandemic has catalysed the emergence of digital solutions in all areas of medicine. Our prior study on the digital health related experiences and opinions of Hungarian physicians highlights the crucial role of age in shaping attitudes towards digital health solutions among medical doctors. Our aim was to examine how under 35-year-old Hungarian physicians relate to digital technologies, the advantages and disadvantages they perceive, and how they would like to incorporate these technologies into their everyday medical practice.

**Methods:**

As part of the “E-physicians and E-patients in Hungary” study, we conducted an online representative survey among medical practitioners in Hungary between July 2021 and May 2022 (*n* = 1,774). The main target group of our research were physicians under 35 years of age: *n* = 399 (25.3%). Besides descriptive statistical analyses, cluster analysis and binary logistic regression were applied to analyse the digital health related attitudes of the young age group.

**Results:**

Our cluster analysis confirmed that younger doctors perceived more advantages (on average 7.07 items vs. 8.52 items) and disadvantages (on average 4.06 vs. 4.42) of digital health solutions. They also demonstrated greater familiarity with (8.27 vs. 9.79) and use of (1.94 vs. 2.66) a broader spectrum of technologies. Proficiency and active utilization of diverse technologies correlates with a more comprehensive understanding of both pros and cons, as well as a more realistic self-assessment of areas of further improvement. Doctors under 35 years express a notable demand for significantly increased incentives, both in terms of knowledge transfer/training and infrastructure incentives. Multivariate analyses revealed that young doctors, compared to their older counterparts, perceived enhanced patient adherence as one of the greatest benefits of digital health solutions. Additionally, young doctors expect that digital health solutions could reduce burnout.

**Conclusion:**

Our results underscore the inevitable transformation of the 21st-century physician role: the success of digital health solutions hinges on active patient involvement and management, which requires proper patient education and professional support in navigating the digital space. Digital health solutions can be a bridge between different generations of doctors, where young people can help their older colleagues navigate the digital world.

## 1 Introduction

The digital transformation of healthcare has become a central issue, profoundly influencing both the current state of health care provision and its future trajectory (1). This shift is reshaping health care organization, human resource management, and even issues of equity and access to care. While it offers solutions to longstanding challenges, it also presents new obstacles. Therefore, it is crucial to understand the factors that encourage both doctors and patients to adopt digital health solutions (2, 3).

Age plays an essential role in digital adaptability. Survey data from the European Commission highlights a substantial increase in daily internet usage among younger populations: in 2021, 95% of young people used the internet daily across Europe, compared to 82% in 2012. By 2023, this figure reached 97% of individuals aged 16–29, compared to 86% of the total population (4). There is evidently a clear correlation between youth and prolonged internet engagement. A pertinent question arises: does increased digital engagement in younger age groups mean they are more likely to embrace digital health solutions?

The concept of Digital Natives, introduced in 2001, remains under debate. In 2009 Prensky suggested that “Digital Wisdom” would be a more fitting term (5). It indicates that as consecutive generations enter the 21st century, nearly everyone will have some digital experience from an early age. This has blurred the divide between “Digital Native” and “Digital Immigrant”. While younger people may be more familiar with digital devices, they still require guidance to use technologiy effectively. Older generations are also rapidly narrowing the gap by using digital innovations and becoming digitally wise. Interestingly, studies show that younger individuals do not necessarily posess better eHealth literacy than older adults (6).

It is generally assumed that young people, including young medical professionals, navigate the digital world with ease. The literature provides evidence in support of this notion. Park and Kwon's systematic review of health-related internet use of children and adolescents show that a high percentage of youth use the internet for health-related purpose (7). Their primarily aim is to find information on daily health related topics. The review also found that many young individuals engege in online health communities and activities such as messaging, networking, and information seeking.

Although the use of digital solutions in healthcare predates COVID-19, it was the imposed isolation and increased medical work load caused by the pandemic that boosted digitalization into mainstream healthcare.

The 2023 survey study of Siragusa et al. highlighted the positive effects of the SARS-CoV-2 crisis on Italian surgical practice (8). One significant development was the increase in the use of teleconsultations for pre and post operation patient management in all forms of surgery (up from 4.1% to 21.6%). Another was the use of digital solutions for diagnostic evaluations (an increase from 16.4% to 42.2%). Finally, there was a big change in surgical professional development in the form of attending e-congresses and participating in online education. Surgeons' personal education online increased from 12.6% (pre-COVID) to 86.6%). The surgeons in the study expressed the desire to continue with teleconsultations and tele-education in the future.

Despite these advancements, relatively little is known about young doctors' (under 35) attitudes to E-health solutions. Research is scarce in the field. According to the American Medical Association's 2022 study on physicians' motivations and expectations for the adaptation of eHealth solutions, the use and acceptance of digital health tools was growing among US based doctors across all ages (9). Although age once influenced enthusiasm for teleHealth according to the results of the 2016 survey, by 2022 this disparity faded, with all age groups recognizing the advantages of digital health solutions. While the 51+ age group has the lowest number of those who see a definite advantage in using digital tools in patient care, they have experienced the largest increase. By 2022 age played no role in the use of remote monitoring in patient care. Similarly, a study from Poland (10) revealed, that 70.4% of the surveyed physicians were familiar with new technologies and rated their eHealth literacy as high, but digital skills decreased significantly with physicians’ age. A comparative study in Hong Kong and Bahrain (11) showed that doctors accept smart phone use in a clinical setting but noted differences by age: 48% of the junior doctors claimed high reliance on smartphones, whereas only 32.3% of the senior doctors reported the same. Correspondingly, a Nigerian survey concluded that doctors under the age of 40 years, particularly Interns, were significantly more likely to perform smartphone-based activities during their hospital work (12). A national survey of the Italian Young Medical Doctors Association (13) found that only a small proportion of young doctors interviewed claimed to have had any significant experience with digital health solutions, like telemedicine tools (22%), big data, omics technology and predictive models, or AI (each 13%), internet of things (6%).

Studies also underscore the need for practical training to keep pace with the digital revolution as most respondents report insufficient preparedness to use digital technologies due to limited exposure to such technologies in medical school (10, 13, 14). While medical students generally hold positive attitudes towards the use of digital health tools in education and patient care, many lack formal training in this field (15, 16).

In our prior study on the eHealth related experiences and opinions of Hungarian physicians (2), we found age to be a significant factor in attitudes towards digital health solutions among medical doctors. The youngest age group displayed the most enthusiasm, while the age group between 35 and 45 was the most intensive user. Our Hungarian population survey on digital health use and attitudes (17) also suggest that age 60 is a critical threshold for both current and planned digital technology use. Additionally, doctors working in the private sector are more prone to recommending websites, applications, or social media sourcse and use telemedicine more often, however, no significant sector-based differences emerged in the use of advanced digital tools like sensors, portable diagnostic devices, AR, VR, robotics, AI and 3Dprinting.

The present study aims to explore the digital health solutions related attitudes of doctors under 35 years, compared to those of older physicians. We investigate the digital tools they prefer to use and the perceived benefits and disadvantages of digital solutions. This topic is critical for both the future of healthcare providers and the patients they serve.

As digital health is a quite broad concept, we have tried to cover as wide a spectrum as possible in our study. The surveyed areas included different activities, like participating in online conferences and trainings, tracking international literature, trends, and data online, telemedicine, smartphone applications, healthcare-related social media, communication with patients, information sharing, home-usable healthcare sensors, smart devices, portable diagnostic devices (e.g., mobile ultrasound, mobile ECG), augmented reality (e.g., surgical practice), use of Virtual Reality (e.g., pain management, psychotherapy), 3D printing (e.g., dental, surgical solutions), Artificial Intelligence solutions in medical decision-making (radiology, pathology, ophthalmology, diagnostic solutions), robotics (e.g., surgical robots, disinfection robots, delivery robots) or anotechnology (e.g., ingestible diagnostic devices).

## 2 Methodology

### 2.1 Recruitment and sample

W e conducted an online survey among medical doctors working in Hungary s part of the “E-physicians and E-patients in Hungary” study (2). The questionnaire was made available online in a self-administered format from July 2021 to May 2022. Our self developed questionnaire is a medical version of the questionnaire used in the population survey (17). The research call with the questionnaire link was emailed to all practising doctors in Hungary (approximately 35.000 people), based on the medical chamber register. All doctors in Hungary had the same chance of being included in the sample, because the Hungarian Medical Chamber (HMC) membership was mandatory for each practising medical doctor.

After the questionnaire was sent out, several reminder emails were sent to increase response rate.

1. **Initial Distribution and Reminders (Summer 2021):** The first wave of invitations and reminders was distributed during the summer of 2021. This period saw lower engagement due to the ongoing pandemic pressures.
2. **Newsletter Campaign (Autumn 2021):** A follow-up campaign was launched in autumn 2021, involving a newsletter sent to HMC members. This aimed to remind and encourage participation among those who might have missed or overlooked the initial emails.
3. **Targeted Email Survey (Spring 2022):** After the COVID-19 epidemic had subsided, a more targeted email survey was conducted in spring 2022. This was done in agreement with the HMC to reach out specifically to physicians who had not yet participated.

A total of 1,774 questionnaires were received, consisting of 1,576 general medical doctors and 198 dentists. Dentist participants were excluded from the present analysis for methodological issues. The target group of our present study are physicians under 35 years of age: *n* = 399 (25.3%).

### 2.2 Measuring instruments

Correction weighting was applied to the responses based on the statistics obtained from the National Register of Practising Medical Doctors. The Directorate of Human Resources Development of the Ministry of Health provided the register. The correction weighting considered factors such as gender, age, and the county where the workplace is, based on the data of the Hungarian Central Statistical Office (18). This correction was necessary due to slight variations in the sample compared to the main distributions of the Register (19). The weight variable had a mean value of 1, a first quartile value of 0.6255, and a third quartile value of 1.1942.

The questionnaire contained items on knowledge, interest, expectation, and use of digital health technologies. The majority of items were on a 5-point Likert scale.

were Sociodemographic data, frequency of internet use for work, knowledge and use of digital health technologies, needs for digital technologies, and positive and negative attitudes towards the use of digital health solutions were the main blocks of the 25-question, 15-min questionnaire.

The questionnaire can be found in the Supplementary Material.

In this research, we looked at digital solutions that our respondents know and use, or are planning to use (in both cases, the top 2 categories of 5-category Lickert scaling questions were considered to be frequently used or intensively used). The internal consistency of the questions was tested using a cronbach's alpha test. The questions on digital technologies (4.1–4.39) were divided into 3 subscales based on “Knowledge”, “Use” and “Willingness to use”.

The Cronbach alpha results for the subscales are. Knowledge (13 items) 0.830, Use (13 items) 0.743 Willingness to apply (13 items): 0.882. These indicators confirmed the reliability of the scales.

We also asked about the perceived advantages and disadvantages of digital health solutions and assessed what help doctors feel they would need to use digital solutions more effectively. The last question was divided into two parts: training aspects (undergraduate, postgraduate training, available protocols, recommendations) and infrastructure aspects (accessibility, affordability, ease of use).

### 2.3 Statistical analysis

Data analysis was performed using IBM Statistics (SPSS 28) (20). The statistical data processing involved examining distributions, conducting cross-tabulation analyses, and performing chi-square tests. Cross-tabulation analyses were conducted using the chi-square test, means were compared using analysis of variance (ANOVA). Logistic regression and K-means analysis were also utilized for deeper context. In our statistical analysis, a significance level of 5% *(p* < 0.05) was applied. Significant associations (*p* < 0.05) are marked in bold in the tables.

The variables used in the analysis were derived from the raw data by aggregating the responses to each question on an individual basis (e.g., assessing the perceived benefits of digital health solutions).

We subsequently ran a K-means cluster analysis, using the variables described above, to test whether two or more groups of digital device users are indeed separated from each other in terms of digital device use.

Finally, we built a multivariate model using logistic regression. The dependent variable was one of the response options of the multiple-choice question asking about the benefits of digital health solutions: it increases patient cooperation and adherence (values: 0 = not selected, 1 = selected). The model was run using a forward conditional variable selection method including a number of variables (age, gender, being a GP or not, the number of advantages and disadvanteges of digital healthcare solutions seen, the number of solutions patients express a need for, the number of digital health solutions the doctor is familiar with, the number of technologies used frequently or on a daily basis, the amount of support that would be necessary (training, protocols, knowledge sharing) to adopt digital healthcare solutions and the amount of support apart from training or knowledge sharing that would be needed to use digital healthcare solutions.

### 2.4 Ethical consideration

This study adhered to ethical guidelines and received approval from TUKEB (Hungarian Scientific Research and Research Ethics Committee) with the reference number IV-10927-1 TUKEB. The research based on anonymized survey data and did not involve interventions or identifiable personal information. Participants provided informed consent by voluntarily completing and submitting the survey online. Confidentiality and anonymity of participants were strictly maintained throughout data collection, analysis, and reporting.

## 3 Results

### 3.1 Demographic profile of the sample

The sample included 399 (25.3%) doctors who were 35 years and under, 845 (53.6%) in the 36–64 age group and 331 (21%) in the 65+ age group. The youngest age group shows a strong female predominance with 62.3% women and 37.8% men. For those aged 65 and over, the gender split is almost even: 47.1% men and 52.9% women. A significant proportion of under-35 s work in inpatient care: 51.6% in hospitals and 29.2% in university clinics. Consequently, the location of their workplace is significantly Budapest (37.5%) and the county seats (41.3%). (See Table 1 for the details).

**Table 1:** Demographic profile.

			35 years old and younger	36–64 years old	65 years old or older
**Gender (*****p*** **<** **0.05)**	**Male**	*n*	**151**	**351**	**156**
		**%**	**37.8%**	**41.5%**	**47.1%**
	**Female**	*n*	**249**	**494**	**175**
		**%**	62.3%	**58.5%**	**52.9%**
	**Total**		**400**	**845**	**331**
T**ype of workplace (*****p*** **< 0.05)**	**University, college**	***n***	**23**	**43**	**13**
		**%**	**5.8%**	**5.1%**	**3.9%**
	**Research institute**	***n***	**3**	**0**	**1**
		**%**	**0.8%**	**0.0%**	**0.3%**
	**Clinic, national specialized hospital**	***n***	116	**89**	**17**
		**%**	**29.2%**	**10.5%**	**5.1%**
	**Hospital**	***n***	**205**	**228**	**54**
		**%**	**51.6%**	**27.0%**	**16.3%**
	**Clinic, other specialist medical institution**	***n***	**5**	**102**	**74**
		**%**	**1.3%**	**12.1%**	**22.4%**
	**General practice**	***n***	**26**	**278**	**110**
		**%**	**6.5%**	**32.9%**	**33.2%**
	**Private healthcare service provider**	***n***	**14**	**64**	**43**
		**%**	**3.5%**	**7.6%**	**13.0%**
	**Multinational company**	***n***	**0**	**6**	**1**
		**%**	**0.0%**	**0.7%**	**0.3%**
	**Hungarian company**	*n*	**3**	**15**	**5**
		**%**	**0.8%**	**1.8%**	**1.5%**
	**Public administration**	***n***	**0**	**6**	**0**
		**%**	**0.0%**	**0.7%**	**0.0%**
	**Health and health-related professional organizations**	***n***	**0**	**1**	**1**
		**%**	**0.0%**	**0.1%**	**0.3%**
	**Other**	***n***	**2**	**13**	**12**
		**%**	**0.5%**	**1.5%**	**3.6%**
	**Total**		**397**	**845**	**331**
**Settlement type of workplace (*****p*** **< 0.05)**	**Capital**	***n***	**149**	**256**	**100**
		**%**	**37.5%**	**30.4%**	**30.3%**
	**County seat**	***n***	**164**	**283**	**86**
		**%**	**41.3%**	**33.6%**	**26.1%**
	**Town**	***n***	**67**	**250**	**119**
		**%**	**16.9%**	**29.7%**	**36.1%**
	**Village, countryside**	*n*	**17**	**54**	**25**
		**%**	**4.3%**	**6.4%**	**7.6%**
	**Total**		**397**	**843**	**330**

Significant correlations are marked in bold.

### 3.2 Outcomes

#### 3.2.1 Familiarity with digital technologies

Looking at individual technologies, following the online literature, apps, various sensors, smart devices, wearable diagnostic devices, AR/VR applications, 3D printing, AI based solutions for robotics and nanotechnology, a significantly higher proportion of the under 35 s indicate that they are more familiar with these technologies (Table 2).

**Table 2:** Familiarity with digital technologies among Hungarian medical doctors, by age group.

		35 years old and younger	36–64 years old	65 years old or older
**Participating in online conferences and trainings**	***n***	**386**	**835**	**310**
	%	**97.0%**	**99.2%**	**94.5%**
**Tracking international literature, trends, and data online**	***n***	**389**	**766**	**260**
	**%**	**97.5%**	**91.1%**	**79.3%**
**Telemedicine, remote visit**	*n*	**338**	**755**	**261**
	**%**	**85.4%**	**89.7%**	**79.1%**
**Smartphone applications, apps**	***n***	**382**	**742**	**236**
	**%**	**95.5%**	**88.4%**	**72.4%**
**Healthcare-related social media, communication with patients, information sharing**	***n***	**321**	**655**	215
	**%**	**80.5%**	**77.8%**	**65.5%**
**Home-usable healthcare sensors, smart devices**	***n***	**386**	**730**	**250**
	**%**	**96.7%**	**86.7%**	**76.5%**
**Portable diagnostic devices (e.g., ultrasound, mobile ECG)**	***n***	**382**	**720**	**255**
	**%**	**96.0%**	**85.8%**	**78.2%**
**Augmented reality (e.g., surgical practice)**	***n***	**204**	**302**	**79**
	**%**	**51.0%**	**35.9%**	**24.2%**
**Use of Virtual Reality (e.g., pain management, psychotherapy)**	***n***	**168**	**276**	**98**
	**%**	**42.0%**	**32.9%**	**29.8%**
**3D printing (e.g., dental, surgical solutions)**	***n***	**285**	**465**	**116**
	**%**	**72.0%**	**55.4%**	**35.5%**
**Artificial intelligence solutions in medical decision-making (radiology, pathology, ophthalmology, diagnostic solutions)**	***n***	**254**	**439**	**131**
	**%**	**63.5%**	**52.1%**	**40.1%**
**Robotics (e.g., surgical robots, disinfection robots, delivery robots)**	***n***	**255**	**416**	**123**
	**%**	**64.1%**	**49.3%**	**37.5%**
**Nanotechnology (e.g., ingestible diagnostic devices)**	***n***	**302**	**546**	**194**
	**%**	**76.1%**	**65.1%**	**59.3%**

Significant correlations are marked in bold.

#### 3.2.2 Use of digital technologies

In addressing the usage of various digital technologies, we utilized responses falling within the range of 4–5 on a Likert scale, where participants indicated frequencies of “often/very often” or daily as the frequency of using different technologies.

Those under 35 showed a significant average in the use of online literature search, apps sensors, smart devices, use of smart devices. On the other hand, the age group 36–64 showed a surplus in the use of teleHealth and social media. (Table 3).

**Table 3:** Usage of digital technologies among Hungarian medical doctors, by age group.

		35 years old and under	36–64 years old	65 years old or older
**Participating in online conferences and trainings**	***n***	**138**	**348**	**81**
	**%**	**34.5%**	**41.3%**	**24.5%**
**Tracking international literature, trends, and data online**	***n***	**238**	**377**	**105**
	**%**	**60.1%**	**44.8%**	**31.9%**
**Telemedicine, remote visit**	***n***	**101**	**382**	**93**
	**%**	**25.4%**	**45.4%**	**28.4%**
**Smartphone applications, apps**	***n***	**256**	**368**	**70**
	**%**	**64.2%**	**43.7%**	**21.4%**
**Healthcare-related social media, communication with patients, information sharing**	***n***	**58**	**175**	**48**
	**%**	**14.5%**	**20.8%**	**14.6%**
**Home-usable healthcare sensors, smart devices**	***n***	**123**	**183**	**53**
	**%**	**30.8%**	**21.7%**	**16.1%**
**Portable diagnostic devices (e.g., ultrasound, mobile ECG)**	***n***	**70**	**139**	**48**
	**%**	**17.5%**	**16.5%**	**14.6%**
**Augmented reality (e.g., surgical practice)**	***n***	**6**	**10**	**5**
	**%**	**1.5%**	**1.2%**	**1.5%**
**Use of Virtual Reality (e.g., pain management, psychotherapy)**	***n***	**9**	**25**	**9**
	**%**	**2.3%**	**3.0%**	**2.7%**
**3D printing (e.g., dental, surgical solutions)**	***n***	**2**	**6**	**0**
	**%**	**0.5%**	**0.7%**	**0.0%**
**Artificial intelligence solutions in medical decision-making (radiology, pathology, ophthalmology, diagnostic solutions)**	***n***	**28**	**37**	**9**
	**%**	**7.0%**	**4.4%**	**2.8%**
**Robotics (e.g., surgical robots, disinfection robots, delivery robots)**	***n***	**4**	**10**	**0**
	**%**	**1.0%**	**1.2%**	**0.0%**
**Nanotechnology (e.g., ingestible diagnostic devices)**	***n***	**2**	**8**	**3**
	**%**	**0.5%**	**0.9%**	**0.9%**

Significant correlations are marked in bold.

#### 3.2.3 Intention of usage of various digital technologies in the next 3 years

For this question, we also used the 4–5 often/very often responses—so we focused on regular users rather than those who just tried them once.

In terms of planned use, young respondents are significantly more likely to want to use online conferencing, apps/sensors, smart devices, mobile diagnostics, AR/VR, 3D technology, robotics, AI and nanotechnology (Table 4).

**Table 4:** Intention of usage of digital technologies in the next 3 years among Hungarian medical doctors, by age group.

		35 years old and younger	36–64 years old	65 years old or older
**Participating in online conferences and trainings**	***n***	**294**	**516**	**139**
	**%**	**73.7%**	**61.4%**	**42.5%**
**Tracking international literature, trends, and data online**	***n***	**353**	**542**	**136**
	**%**	**89.6%**	**64.8%**	**41.2%**
**Telemedicine, remote visit**	***n***	**197**	**445**	**106**
	**%**	**49.3%**	**53.0%**	**32.3%**
**Smartphone applications, apps**	***n***	**317**	**475**	**93**
	**%**	**79.6%**	**56.7%**	**28.6%**
**Healthcare-related social media, communication with patients, information sharing**	***n***	**128**	**257**	**65**
	**%**	**32.7%**	**30.7%**	**20.0%**
**Home-usable healthcare sensors, smart devices**	***n***	**267**	**374**	**84**
	**%**	**67.1%**	**44.4%**	**25.7%**
**Portable diagnostic devices (e.g., ultrasound, mobile ECG)**	***n***	**309**	**373**	**90**
	**%**	**77.8%**	**44.7%**	**27.6%**
**Augmented reality (e.g., surgical practice)**	***n***	**138**	**114**	**10**
	**%**	**34.6%**	**13.5%**	**3.1%**
**Use of Virtual Reality (e.g., pain management, psychotherapy)**	***n***	**109**	**105**	**21**
	**%**	**27.3%**	**12.5%**	**6.4%**
**3D printing (e.g., dental, surgical solutions)**	***n***	**155**	**131**	**12**
	**%**	**38.8%**	**15.6%**	**3.7%**
**Artificial intelligence solutions in medical decision-making (radiology, pathology, ophthalmology, diagnostic solutions)**	***n***	**172**	**240**	**34**
	**%**	**43.1%**	**28.6%**	**10.4%**
**Robotics (e.g., surgical robots, disinfection robots, delivery robots)**	***n***	**137**	**139**	**17**
	**%**	**34.4%**	**16.6%**	**5.2%**
**Nanotechnology (e.g., ingestible diagnostic devices)**	***n***	**172**	**197**	**39**
	**%**	**43.1%**	**23.4%**	**11.9%**

Significant correlations are marked in bold.

#### 3.2.4 Perceived need to facilitate the use of digital solutions

A significantly higher proportion of doctors under 35 indicated that financial incentives, accessible professional materials, availability of technologies, evidence-based studies, clarification of legal-ethical issues, data security, existence of different professional protocols and patient engagement would be a prerequisite for the higher uptake of digital solutions.

It can also be seen that on average, doctors under 35 years cited the need for more incentives, both in terms of knowledge transfer/training and infrastructure incentives (Table 5).

**Table 5:** Perceived need for using digital technologies among Hungarian medical doctors, by age group.

		35 years and under	36–64 years old	65 years old or older
**Financial incentives (e.g., support for acquiring certain tools)**	***n***	**303**	**571**	**202**
	**%**	**75.9%**	**67.6%**	**61.0%**
**Postgraduate training**	***n***	**94**	**300**	**158**
	**%**	**23.5%**	**35.5%**	**47.7%**
Other training opportunities	*n*	179	367	142
	%	44.9%	43.4%	42.9%
**Accessible professional materials (documents, online training, etc.)**	***n***	**287**	**493**	**172**
	**%**	**71.9%**	**58.3%**	**52.0%**
**Availability and accessibility of technologies**	***n***	**328**	**574**	**165**
	**%**	**82.0%**	**67.9%**	**49.8%**
Recommendations from colleagues	*n*	69	129	40
	%	17.3%	15.3%	12.1%
**Evidence-based research**	***n***	**221**	**320**	**91**
	**%**	**55.3%**	**37.9%**	**27.5%**
**Ethical and legal regulations**	***n***	**255**	**484**	**150**
	**%**	**63.9%**	**57.3%**	**45.3%**
**Professional protocols**	***n***	**298**	**508**	**189**
	**%**	**74.7%**	**60.2%**	**57.1%**
**Data security protocols**	***n***	**247**	**420**	**130**
	**%**	**61.8%**	**49.7%**	**39.3%**
**Dedicated time within working hours**	***n***	**294**	**600**	**166**
	**%**	**73.7%**	**71.0%**	**50.2%**
**Patient commitment and increased collaboration**	***n***	**209**	**355**	**145**
	**%**	**52.4%**	**42.0%**	**43.8%**

Significant correlations are marked in bold.

#### 3.2.5 Perceived benefits of different digital solutions by young doctors—a paradigm shift

Overall, younger doctors tend to mention more benefits. A significantly higher proportion reported improved efficiency, improved diagnostic skills, reduced burnout, increased patient adherence, better patient involvement in the healing process, increased patient cooperation, more comfort, time saving, improved quality of care and reduced rate of malpractice. Also a higher proportion of young doctors think that using digital tools will bring extra income to their practice (Table 6).

**Table 6:** Perceived benefits of digital technologies among Hungarian medical doctors, by age group.

		35 years old and under	36–64 years old	65 years old or older
**Improved efficiency**	***n***	**313**	**578**	**200**
	**%**	**78.4%**	**68.4%**	**60.4%**
Enhanced safety	*n*	122	227	96
	%	30.6%	26.9%	29.0%
**Improved diagnostic capabilities**	***n***	**199**	**349**	**145**
	**%**	**49.9%**	**41.3%**	**43.8%**
**Reduced burnout**	***n***	**147**	**233**	**63**
	**%**	**36.8%**	**27.6%**	**19.0%**
**Increased patient adherence and collaboration**	***n***	**265**	**457**	**139**
	**%**	**66.3%**	**54.1%**	**42.0%**
**Convenient**	***n***	**331**	**563**	**187**
	**%**	**83.0%**	**66.6%**	**56.5%**
**Reduced the number of in-person doctor-patient meetings**	***n***	**209**	**547**	**178**
	**%**	**52.4%**	**64.7%**	**53.8%**
**Save time for the doctor**	***n***	**271**	**473**	**152**
	**%**	**67.8%**	**56.0%**	**45.9%**
**Save time for the patient**	***n***	**282**	**562**	**170**
	**%**	**70.7%**	**66.5%**	**51.4%**
**Enable faster access to healthcare**	***n***	**207**	**453**	**147**
	**%**	**51.9%**	**53.6%**	**44.4%**
**Make your work more efficient**	***n***	**236**	**418**	**126**
	**%**	**59.1%**	**49.5%**	**38.1%**
**Engage patients more actively in their own healing process**	***n***	**216**	**403**	**117**
	**%**	**54.1%**	**47.7%**	**35.3%**
**Improve the quality of care**	***n***	**187**	**309**	**104**
	**%**	**46.9%**	**36.6%**	**31.4%**
**Reduce the likelihood of errors**	***n***	**103**	**147**	**57**
	**%**	**25.8%**	**17.4%**	**17.2%**
**Generate additional income for doctors**	***n***	**54**	**65**	**10**
	**%**	**13.5%**	**7.7%**	**3.0%**
**Increase patient satisfaction**	***n***	**251**	**400**	**115**
	**%**	**62.9%**	**47.3%**	**34.7%**
**Improve doctor-patient communication**	***n***	**193**	**353**	**158**
	**%**	**48.4%**	**41.8%**	**47.7%**

Significant correlations are marked in bold.

##### 3.2.5.1 Perceived disadvantages of using digital tools

Doctors under 35 years also mention more disadvantages on average than the other two age groups. They cite significantly higher rates of overdiagnosis, patients misinterpreting the data they share, technological errors that jeopardise patient recovery, data protection problems, and increased administrative burden (Table 7).

**Table 7:** Perceived disadvantages of digital technologies among Hungarian medical doctors, by age group.

		35 years old and under	36–64 years old	65 years old or older
Decreased quality of care	*n*	115	268	96
	%	28.8%	31.7%	29.0%
Frustration among patients	*n*	67	183	75
	%	16.8%	21.7%	22.7%
**Potential for overdiagnosis**	***n***	**223**	**259**	**100**
	**%**	**55.9%**	**30.7%**	**30.2%**
**Misinterpretation of shared health data by patients**	***n***	**287**	**522**	**221**
	**%**	**71.9%**	**61.8%**	**66.8%**
Increased possibility of misunderstandings in doctor-patient communication	*n*	188	433	163
	%	47.1%	51.2%	49.2%
**Faulty technology jeopardizing patient recovery**	***n***	**126**	**196**	**79**
	**%**	**31.6%**	**23.2%**	**23.9%**
**Compromised confidentiality of patient data**	***n***	**191**	**367**	**116**
	**%**	**47.9%**	**43.4%**	**35.0%**
**Increased administrative burdens on doctors**	***n***	**229**	**435**	**159**
	**%**	**57.4%**	**51.5%**	**48.0%**
Additional costs for practices	*n*	143	302	109
	%	35.8%	35.7%	32.9%
Limited patient proficiency in using digital technologies, placing a burden on the treating physician	*n*	229	433	189
	%	57.4%	51.2%	56.9%
Increased likelihood of burnout	*n*	42	100	31
	%	10.5%	11.8%	9.4%

Significant correlations are marked in bold.

#### 3.2.6 Cluster analysis

In the next step, we ran a K-means cluster analysis on the data to investigate whether the younger age group is indeed distinct from older doctors in their attitudes towards digital health technologies. The final model with two clusters was generated as a result of the run, where the distribution of item numbers is half and half, with 786 people in the first cluster and 788 in the second cluster. The final cluster centres table shows that the two groups differ significantly and spectacularly in age (63.29 vs. 36.35), and there are also differences in the other variables included in the model. Younger doctors perceive more advantages (7.07 vs. 8.52) and disadvantages (4.06 vs. 4.42) of digital health solutions, are more familiar with (8.27 vs. 9.79) and use (1.94 vs. 2.66) more technologies. There is no significant difference in the needs perceived by patients (3.84 vs. 3.90) and in the amount of things they would need training or knowledge transfer for (2.43 vs. 2.63). Other differences (no training or knowledge transfer) are also visible between the two groups (3.18 vs. 4.02) (Table 8).

**Table 8:** Clusters of Hungarian medical doctors, by digital technology use.

	Final cluster centers	
	1	2
How many advantages do digital healthcare solutions have?	7.07	8.52
How many disadvantages do digital healthcare solutions have?	4.06	4.42
How many solutions do your patients express a need for?	3.84	3.90
How many digital health solutions are known by you?	8.27	9.79
How many technologies do you use frequently or on a daily basis?	1.94	2.66
Age of respondent	63.29	36.35
How much support would be necessary (training, protocols, knowledge sharing) for you to adopt digital healthcare solutions?	2.43	2.63
Apart from training or knowledge sharing, how much support would be needed for you to use digital healthcare solutions?	3.18	4.02
*n*	786	788

#### 3.2.7 Logistic regression model for patient adherence and use of digital solutions

As a last step of our analysis, we ran a multivariate model with logistic regression, where the dependent variable was the question of increasing patient adherence (0 = not marked, 1 = marked). The final logistic regression model included 5 explanatory variables (Nagelkerke R-square = 0.261).

The age of the responding physician is a significant explanatory factor in the model, with an odds ratio of 0.991 CI: 0,983–0,998) so the older someone is, the less likely they are to think that digital health solutions increase patient adherence. The gender variable is also significant, with a positive odds ratio, so women are more likely to think that it increases adherence.

Other significant explanatory variables are the number of technologies used often or every day, the number of needed technologies (not knowledge, training or knowledge transfer, but infrastructure), and perceived benefits (other than increasing patient adherence). These variables are all odds ratios above 1, so the correlation is positive, i.e., the more technology used, the more infrastructure items would be needed, and the more benefits perceived from digital health solutions, the more likely they are to think it increases patient adherence (Table 9).

**Table 9:** Logistic regressions to determine the factors associated with increasing patient adherence.

Nagelkerke R-square = 0,261						
	B	S.E.	Wald	df	Sig.	Exp(B)
Age of respondent	−0.009	0.004	6.070	1	0.014	0.991
Gender of respondent (reference: 1 = male; 2 = female)	0.336	0.116	8.459	1	0.004	1.399
How many technologies do you use frequently or on a daily basis?	0.072	0.033	4.695	1	0.030	1.075
How much support would be necessary (training, protocols, knowledge sharing) for you to adopt digital healthcare solutions?	0.167	0.037	20.117	1	0.000	1.182
Apart from training or knowledge sharing, how much support would be needed for you to use digital healthcare solutions?	0.220	0.019	138.769	1	0.000	1.246
Constant	−1.843	0.296	38.883	1	0.000	0.158

## 4 Discussion

The WHO report “Global strategy on digital health 2020–2025”, showed that digital technologies were essential for sustainable health systems and universal health coverage (21). Digital health, or eHealth, uses information and communication technologies and networks to manage, deliver and optimise patient care and health services. According to WHO data there are currently 5–7 million health workers missing from health systems worldwide (22). One possible alternative of addressing the health workforce shortage is the increasing use of digital technologies in various care settings.

A systematic review of 123 studies, encompassing data from approximately 250,000 healthcare providers globally, revealed that the adoption of mobile technologies, telemedicine, and various digital tools for clinical decision support has positively impacted the performance, mental well-being, skills, and competencies of health workers (23).

In this context, a key question is how young doctors relate to digital technologies, what advantages and disadvantages they see in these possibilities and how they would like to incorporate their use into their everyday medical practice.

Digital transformation in the health sector is not a simple matter of technical change, but requires adaptive changes in human attitudes and skills, too (24). The past 20 years have seen the emergence of e-patients and e-physicians. E-patients are active partners in the care they receive while manifesting the power of the participatory medicine model. The “e” stands for “electronic”, “equipped”, “enabled” “empowered”, “engaged” and “expert” (25). On the other side, e-physicians are “electronic” as they use digital technologies in their practice with ease. They are “equipped” because they have digital health technologies at their disposal. They are “enabled” by regulations and guidelines and “empowered” by technologies that support their job. They are “engaged” in as much as they have empathy to understand the feelings and point of view of patients, give them relevant feedback and involve them throughout the whole healing process. Finally, they are “experts” in using technologies in their practice and knowing the best and most reliable and trustworthy sources and technologies. As we can see from these definitions, being an e-patient or e-physician goes beyond digital proficiency, it is also an attitude and an orientation (24, 26).

Digital health solutions make new approaches of care possible by moving diagnosis, treatment, and prevention out of the clinic s into the everyday settings of people's lives (27). From this perspective, the dynamics of the doctor- patient relationship in the 21st century are influenced by the possibilities inherent in digital solutions and the democratization of health care. This phenomenon is best captured by the phrase of *Participatory Medicine*. Participatory Medicine is a movement in which patients and health professionals actively collaborate and encourage one another as full partners in healthcare. Hood and Auffray defined it in 2013 as “an approach of cooperative health care that actively and continuously involves patients and other stakeholders (i.e., healthcare providers and caregivers), across the continuum of care” (28).

Participatory medicine is an attitude. It is a considerable shift from the parentalistic paternalistic medical model characteristic of the 20th century and before. Patients are accepted as partners and joint decision making is favoured. It started before digitalisation in healthcare with the patients' rights movement and the legal requirement of informed consent. Digitalisation suits this model as in essence it facilitates the information flow towards patients. They are given access to Electronic Health Records, they have tools to monitor their health status at home with wearable devices. They have easier access to their healthcare provider through telemedicine. They can gain health related information from online sources and provide and receive peer support in online patient communities (29).

Our cluster analysis confirmed the hypothesis that younger individuals exhibit a greater familiarity with digital solutions. Specifically, doctors under the age of 35 demonstrated a significantly higher level of familiarity with utilization of and future interest in digital technology. Proficiency in multiple technologies correlated with a more informed understanding of both advantages and disadvantages, fostering a realistic assessment of the need for further improvement. Our results underscore a pronounced demand for both training and infrastructure in the use of digital health-related technologies. It is noteworthy that in comparison to their elder counterparts, young doctors identify enhanced patient adherence, increased patient involvement in the healing process and heightened patient cooperation as the greatest benefits of digital health solutions. In our multivariate analysis, a clear connection emerged between young doctors, active patient involvement in the treatment process—and the pivotal role played by digital technologies in facilitating this engagement Additionally, our results highlight the indispensable nature of a proper patient-provider relationship for the successful participation and adherence process which based on mutual communication, data sharing and shared decison making (30–32).

Digital health solutions can potentially enhance patient adherence (33–35), although relevant studies are small-scale and limited. More work is needed to identify the most effective digital health interventions to further quality health care provision (34, 36–40).

Our multivariate analysis also revealed that women are more likely than their male counterparts to believe that digital health solutions have a significant role to play in improving patient adherence. Significant attention has been devoted to researching how gender of both patients and physicians are related to the overall patient experience and specific elements of the patient-physician relationship (41).

The role of digital health solutions in patient empowerment is supported by a large body of research (42–44) but little research has focused at the impact of the gender of the patient empowerment.

Another significant result of our study is that young doctors think that digital technologies may potentially be able to reduce burnout. According to the study of Rotenstein et al. nearly half of all healthcare workers suffered from burnout during the COVID-19 pandemic (45). The 2023 Physician Burnout & Depression Report of Medscape entitled “I Cry but No One Cares,” is a survey of more than 9,100 doctors representing 29 specialties. This study concludes that 53% of physicians experienced burnout the previous year, which is a six percent increase from 2021. Nearly a quarter of the surveyed physicians reported having been depressed, up from 15% in 2018 (46). Our pre-pandemic Hungarian data shows that medium or high level personal accomplishment was present in 75.9% of surveyed doctors and emotional exhaustion in 58% The moderate level depersonalization subscale was 53%. All 3 aspects showed association with being under 35 years old, working in in-patient care, working shifts and multiple workplaces (47).

Evidence is still scarce, but it tends to point to the burnout-reducing potential that digital health may have. Nevertheless, we must say that initially EHR was demonstrated to negatively influence clinician well-being, contributing to clinician burnout (48). Digital health solutions can improve job satisfaction and reduce burnout supporting clinical decision-making and reducing administrative tasks (49). Digital solutions can save time, minimize repetition, improve team-work and reduce unnecessary visits (50). One of the main sources of burnout in healthcare is the overwhelming amount of administrative and repetitive tasks. For example, AI can take over administrative tasks, using natural language processing, computer vision, speech recognition, and machine learning to streamline workflow. The uncertainty and complexity of clinical decisions may also contribute to burnout. AI can ease this as a decision aid using data analytics and predictive modeling. Chatbots, virtual assistants, and teleHealth platforms enable better interaction with healthcare workers and patients. These AI-driven technologies may improve efficiency and support healthcare professionals, contributing to their well-being and combating burnout (51–53).

## 5 Conclusions

Our results show the inevitable transformation of the 21st century physician's role: the success of digital health solutions requires the active involvement and management of patients, which demands proper patient education and professional support in navigating the digital space (54). As for physicians, it is important to provide guidance for patients in the DH world, especially in the areas of creditibility and proper use. This raises the need to incorporate digital health skills as an important part of medical and professional training. The 2024 position paper of the European Federation of Internal Medicine (EFIM) supports this notion (55). Having used SWOT analysis and the DELPHI method to reach their conclusion, the panel of experts consulted strongly advocates the development and application of telemedicine and digital technologies in healthcare. For this aim they emphesise the importance of professional development of eHealth competencies in the healthcare and medical workforce. They also stress the need for the development of consensus on care models or standardized protocols among European Internal Medicine specialists regarding telemedicine use.

Another important trend emerged from our findings: young doctors' needs and skills in digital technologies can be an important reference point for older colleagues. Digital health solutions can be a bridge between different generations of doctors, where young people can help their older colleagues navigate the digital world.

### 5.1 Research implications

The implications of digital health extend across theoretical, practical, and social dimensions, calling for an integrated approach that combines technology, education, and systemic innovation to achieve sustainable progress in healthcare.

The results of our study can be summarised in this theoretical framework as follows:

### 5.2 Theoretical implications

Digital health introduces a paradigm shift in healthcare emphasizing mutual care and challenging traditional hierarchies. The emergence of “e-patients” and “e-physicians” highlights the need for engagement and expertise, reflecting a systemic integration of technology and human attitudes. Digital tools address global challenges like workforce shortages and burnout by optimizing workflows and decision-making.

### 5.3 Practical implications

Digital health solutions enhance clinical efficiency and reduce administrative burdens through tools like telemedicine, AI-driven solutions, and mobile apps. Young doctors play a pivotal role in emphasizing the advantages of enhanced patient adherence and collaboration. Successful integration of digital health solutions depends on comprehensive training, adequate infrastructure, and effective patient education to maintain credibility and achieve full potential.

### 5.4 Social implications

Digital health reshapes doctor-patient relationships by promoting transparency and collaboration, as seen in Participatory Medicine. Young doctors help bridge generational divides, aiding older colleagues in adapting to new technologies.

### 5.5 Strengths and limitations

The strength of our research is that it analyses the proficiency and attitudes towards digital technologies of Hungarian doctors under 35 years of age in a representative sample. There is very limited published data on the digital health related attitudes of the young generation of physicians in the international literature. An additional strength is that our results are comparable with an equally representative sample of senior doctors. However, a limitation of our research is that we did not define the concept of patient adherence in our survey. It is also a limitation that burnout was measured subjectively, and no instrument was used for this. Another limitation is that a significant proportion of young doctors' work in inpatient care and urban areas which makes it difficult to compare different fields of care. One final limitation is that we have examined several different digital tools. This makes it difficult to compare knowledge, usage patterns and opportunities.

## Data Availability

The raw data supporting the conclusions of this article will be made available by the authors, without undue reservation.

## References

[1] Expert Panel on effective ways of investing in Health. Assessing the impact of digital transformation of health services. Available online at: https://health.ec.europa.eu/system/files/2019-11/022_digitaltransformation_en_0.pdf (Accessed January 02, 2024).

[2] Girasek E Boros J Döbrössy B Győrffy Z. E-orvosok Magyarországon: Digitális egészséggel kapcsolatos tapasztalatok és vélemények a hazai orvosok körében [E-physicians in Hungary: experiences and opinions related to digital health among Hungarian physicians]. Orv Hetil. (2023) 164(4):132–9. 10.1556/650.2023.32686 36709435

[3] Stoumpos AI Kitsios F Talias MA. Digital transformation in healthcare: technology acceptance and its applications. Int J Environ Res Public Health. (2023) 20(4):3407. 10.3390/ijerph20043407 36834105 PMC9963556

[4] Eurostat. Young people-digital world. Statistics Explained (2024). Available online at: https://ec.europa.eu/eurostat/statistics-explained/index.php?title=Youngpeople-digitalworld#Adigitalage_gap (Accessed December 12, 2024).

[5] Prensky MH. Sapiens digital: from digital immigrants and digital natives to digital wisdom. Innovate J Online Educ. (2009) 5(3):1. https://nsuworks.nova.edu/innovate/vol5/iss3/1

[6] Stellefson M Hanik B Chaney B Chaney D Tennant B Chavarria EA. Ehealth literacy among college students: a systematic review with implications for eHealth education. J Med Internet Res. (2011) 13(4):e102. 10.2196/jmir.1703 22155629 PMC3278088

[7] Park E Kwon M. Health-related internet use by children and adolescents: systematic review. J Med Internet Res. (2018) 20(4):e120. 10.2196/jmir.7731 29615385 PMC5904452

[8] Siragusa L Angelico R Angrisani M Zampogna B Materazzo M Sorge R How future surgery will benefit from SARS-COV-2-related measures: a SPIGC survey conveying the perspective of Italian surgeons. Updates Surg. (2023) 75(6):1711–27. 10.1007/s13304-023-01613-5 37578735 PMC10435629

[9] AMA Digital Health Research. Physicians’ motivations and key requirements for adopting digital health. Adoption and attitudinal shifts from 2016 to 2022. Available online at: https://www.ama-assn.org/system/files/ama-digital-health-study.pdf (Accessed January 02, 2024).

[10] Burzyńska J Bartosiewicz A Januszewicz P. Dr. Google: physicians-the web-patients triangle: digital skills and attitudes towards eHealth solutions among physicians in South Eastern Poland-A cross-sectional study in a pre-COVID-19 era. Int J Environ Res Public. (2023) 20(2):978. 10.3390/ijerph20020978 PMC9858975 36673740

[11] Nair AA Afroz S Ahmed BU Ahmed UU Foo CC Zaidan H Smartphone usage among doctors in the clinical setting in two culturally distinct countries: cross-sectional comparative study. JMIR Mhealth Uhealth. (2021) 9(5):e22599. 10.2196/22599 33970119 PMC8145086

[12] Yahya H. Healthcare-related smartphone use among doctors in hospitals in Kaduna, Nigeria—a survey. Niger J Clin Pract. (2019) 22(7):897–905. 10.4103/njcp.njcp_454_18 31293252

[13] Casà C Marotta C Di Pumpo M Cozzolino A D'Aviero A Frisicale EM COVID-19 and digital competencies among young physicians: are we (really) ready for the new era? A national survey of the Italian young medical doctors association. Ann Ist Super Sanita. (2021) 57(1):1–6. 10.4415/ANN_21_01_01 33797398

[14] Zainal H Xiaohui X Thumboo J Yong FK. Exploring the views of Singapore junior doctors on medical curricula for the digital age: a case study. PLoS One. (2023) 18(3):e0281108. 10.1371/journal.pone.0281108 36862708 PMC9980755

[15] Ma M Li Y Gao L Xie Y Zhang Y Wang Y The need for digital health education among next-generation health workers in China: a cross-sectional survey on digital health education. BMC Med Educ. (2023) 23(1):541. 10.1186/s12909-023-04407-w 37525126 PMC10388510

[16] Poncette AS Glauert DL Mosch L Braune K Balzer F Back DA. Undergraduate medical competencies in digital health and curricular module development: mixed methods study. J Med Internet Res. (2020) 22(12):1–14. 10.2196/22161 PMC7661229 33118935

[17] Girasek E Boros J Döbrössy B Susánszky A Győrffy ZS. E-páciensek Magyarországon: Digitális egészséggel kapcsolatos ismeretek, szokások egy országos reprezentatív felmérés tükrében [E-patients in Hungary: digital health use and attitudes based on a representative nationwide survey]. Orv Hetil. (2022) 163(29):1159–65. 10.1556/650.2022.32512 35895447

[18] Lavrakas PJ. Encyclopedia of Survey Research Methods. Thousand Oaks, CA: SAGE Publications Ltd. (2008). 10.4135/9781412963947

[19] National Directorate General for Hospitals. Information about the administrative break. Available online at: https://enkk.hu/index.php/en/ (Accessed January 02, 2024).

[20] IBM Corporation. IBM SPSS Statistics for Windows, Version 27.0. Armonk, NY: IBM Corporation (2020).

[21] World Health Organization. World Health Organization Global Strategy on Digital Health 2020–2025. Geneva: World Health Organization (2021). https://www.who.int/docs/default-source/documents/gs4dhdaa2a9f352b0445bafbc79ca799dce4d.pdf

[22] World Health Organization. WHO Global Strategy on Human Resources for Health: Workforce 2030. Geneva: World Health Organization (2020). https://www.who.int/publications/i/item/9789241511131

[23] Borges do Nascimento IJ Abdulazeem HM Vasanthan LT Martinez EZ Zucoloto ML Østengaard L The global effect of digital health technologies on health workers’ competencies and health workplace: an umbrella review of systematic reviews and lexical-based and sentence-based meta-analysis. Lancet Digit Health. (2023) 5(8):e534–44. 10.1016/S2589-7500(23)00092-4 37507197 PMC10397356

[24] Meskó B Drobni Z Bényei É Gergely B Győrffy Z. Digital health is a cultural transformation of traditional healthcare. Mhealth. (2017) 3:38. 10.21037/mhealth.2017.08.07 29184890 PMC5682364

[25] Ferguson T Frydman G. The first generation of e-patients. Br Med J. (2004) 328(7449):1148–9. 10.1136/bmj.328.7449.1148 15142894 PMC411079

[26] Mesko B Győrffy Z. The rise of the empowered physician in the digital health era: viewpoint. J Med Internet Res. (2019) 21(3):e12490. 10.2196/12490 30912758 PMC6454334

[27] Schueller SM. Grand challenges in human factors and digital health. Front Digit Health. (2021) 3:635112. 10.3389/fdgth.2021.635112 34713105 PMC8522014

[28] Hood L Auffray C. Participatory medicine: a driving force for revolutionizing healthcare. Genome Med. (2013) 5(12):110. 10.1186/gm514 24360023 PMC3978637

[29] Menichetti J Libreri C Lozza E Graffigna G. Giving patients a starring role in their own care: a bibliometric analysis of the on-going literature debate. Health Expect. (2016) 19:516–26. 10.1111/hex.12299 25369557 PMC5055237

[30] Charles C Gafni A Whelan T. Shared decision-making in the medical encounter: what does it mean? (or it takes at least two to tango). Soc Sci Med. (1997) 44:681–92. 10.1016/s0277-9536(96)00221-3 9032835

[31] Deniz S Akbolat M Çimen M Ünal Ö. The mediating role of shared decision-making in the effect of the patient–physician relationship on compliance with treatment. J Patient Exp. (2021) 8:23743735211018066. 10.1177/23743735211018066 34179444 PMC8205395

[32] Iyawa GE Herselman M Botha A. Digital health innovation ecosystems: from systematic literature review to conceptual framework. Procedia Comput. Sci. (2016) 100:244–52. 10.1016/j.procs.2016.09.149

[33] Ridho A Alfian SD van Boven JFM Levita J Yalcin EA Le L Digital health technologies to improve medication adherence and treatment outcomes in patients with Tuberculosis: systematic review of randomized controlled trials. J Med Internet Res. (2022) 24(2):e33062. 10.2196/33062 35195534 PMC8908199

[34] O'Hara DV Yi TW Lee VW Jardine M Dawson J. Digital health technologies to support medication adherence in chronic kidney disease. Nephrology (Carlton). (2022) 27(12):917–24. 10.1111/nep.14113 36176176 PMC9828762

[35] Babel A Taneja R Mondello Malvestiti F Monaco A Donde S. Artificial intelligence solutions to increase medication adherence in patients with non-communicable diseases. Front. Digit Health. (2021) 3:669869. 10.3389/fdgth.2021.669869 34713142 PMC8521858

[36] Brands MR Gouw SC Beestrum M Cronin RM Fijnvandraat K Badawy SM. Patient-centered digital health records and their effects on health outcomes: systematic review. J Med Internet Res. (2022) 24(12):e43086. 10.2196/43086 36548034 PMC9816956

[37] Jakob R Harperink S Rudolf AM Fleisch E Haug S Mair JL Factors influencing adherence to mHealth apps for prevention or management of noncommunicable diseases: systematic review. J Med Internet Res. (2022) 24(5):e35371. 10.2196/35371 35612886 PMC9178451

[38] Gandapur Y Kianoush S Kelli HM Misra S Urrea B Blaha MJ The role of mHealth for improving medication adherence in patients with cardiovascular disease: a systematic review. Eur Heart J Qual Care Clin Outcomes. (2016) 2(4):237–44. 10.1093/ehjqcco/qcw018 29474713 PMC5862021

[39] Hamine S Gerth-Guyette E Faulx D Green BB Ginsburg AS. Impact of mHealth chronic disease management on treatment adherence and patient outcomes: a systematic review. J Med Internet Res. (2015) 17(2):e52. 10.2196/jmir.3951 25803266 PMC4376208

[40] Anglada-Martinez H Riu-Viladoms G Martin-Conde M Rovira-Illamola M Sotoca-Momblona JM Codina-Jane C. Does mHealth increase adherence to medication? Results of a systematic review. Int J Clin Pract. (2015) 69(1):9–32. 10.1111/ijcp.12582 25472682

[41] Takeshita J Wang S Loren AW Mitra N Shults J Shin DB Association of racial/ethnic and gender concordance between patients and physicians with patient experience ratings. JAMA Netw Open. (2020 Nov 2) 3(11):e2024583. 10.1001/jamanetworkopen.2020.24583 33165609 PMC7653497

[42] Saukkonen P Elovainio M Virtanen L Kaihlanen A Nadav J Lääveri T The interplay of work, digital health usage, and the perceived effects of digitalization on Physicians’ work: network analysis approach. J Med Internet Res. (2022) 24(8):e38714. 10.2196/38714 35976692 PMC9434392

[43] Dang A Arora D Rane P. Role of digital therapeutics and the changing future of healthcare. J Family Med Prim Care. (2020) 5:2207–13. 10.4103/jfmpc.jfmpc_105_20 PMC7380804 32754475

[44] Győrffy Z Radó N Mesko B. Digitally engaged physicians about the digital health transition. PLoS One. (2020) 15(9):e0238658. 10.1371/journal.pone.0238658 32986733 PMC7521720

[45] Rotenstein LS Brown R Sinsky C Linzer M. The association of work overload with burnout and intent to leave the job across the healthcare workforce during COVID-19. J Gen Intern Med. (2023) 38(8):1920–7. 10.1007/s11606-023-08153-z 36959522 PMC10035977

[46] Medscape. ‘I Cry but No One Cares’: Physician Burnout & Depression Report (2023). Available online at: https://www.medscape.com/slideshow/2023-lifestyle-burnout-6016058 (Accessed January 04, 2024).

[47] Győrffy ZS Girasek E. [Burnout among Hungarian physicians. Who are the most jeopardized?]. Orv. Hetil. (2015) 156(14):564–70. 10.1556/oh.2015.30121 25819150

[48] Wosny M Strasser LM Hastings J. Experience of health care professionals using digital tools in the hospital: qualitative systematic review. JMIR Hum Factors. (2023) 10:e50357. 10.2196/50357 37847535 PMC10618886

[49] Price T. How technology can ease provider burnout and improve patients’ health care experience (2023). Available online at: https://www.medicaleconomics.com/view/how-technology-can-ease-provider-burnout-and-improve-patients-health-care-experience (Accessed January 04, 2024).

[50] Thomas Craig KJ Willis VC Gruen D Rhee K Jackson GP. The burden of the digital environment: a systematic review on organization-directed workplace interventions to mitigate physician burnout. J Am Med Inform Assoc. (2021) 28(5):985–97. 10.1093/jamia/ocaa301 33463680 PMC8068437

[51] Akinrinmade AO Adebile TM Ezuma-Ebong C Bolaji K Ajufo A Adigun AO Artificial intelligence in healthcare: perception and reality. Cureus. (2023) 15(9):e45594. 10.7759/cureus.45594 37868407 PMC10587915

[52] Laker B Currell E. ChatGPT: a novel AI assistant for healthcare messaging-a commentary on its potential in addressing patient queries and reducing clinician burnout. BMJ Lead. (2024) 8(2):147–8. 10.1136/leader-2023-000844 37751926

[53] Gandhi TK Classen D Sinsky CA Rhew DC Vande Garde N Roberts A How can artificial intelligence decrease cognitive and work burden for front line practitioners? JAMIA Open. (2023) 6(3):ooad079. 10.1093/jamiaopen/ooad079 37655124 PMC10466077

[54] Butcher CJ Hussain W. Digital healthcare: the future. Future Healthc J. (2022) 9(2):113–7. 10.7861/fhj.2022-0046 35928188 PMC9345235

[55] Pietrantonio F Florczak MM Kuhn S Kärberg K Leung T Said Criado I Applications to augment patient care for internal medicine specialists: a position paper from the EFIM working group on telemedicine, innovative technologies & digital health. Front Public Health. (2024) 12:1370555. 10.3389/fpubh.2024.1370555 39005984 PMC11239350

